# Performance of PROPELLER FSE T_2_WI in reducing metal artifacts of material porcelain fused to metal crown: a clinical preliminary study

**DOI:** 10.1038/s41598-022-12402-2

**Published:** 2022-05-19

**Authors:** Wenjin Li, Jing Shi, Wenjin Bian, Jianting Li, Xiaoqing Chen, Juan Feng, Jiali Yu, Jun Wang, Jinliang Niu

**Affiliations:** 1 Departments of Stomatology, The Second Hospital of Shanxi Medical University, Taiyuan, Shanxi China; 2 Departments of Stomatology, The Second Hospital of Tianjin Medical University, Tianjin, China; 3 Department of Medical Imaging, Shanxi Medical University, Taiyuan, Shanxi China; 4 Department of Radiology, The Second Hospital of Shanxi Medical University, NO.382 Wuyi Road, Taiyuan, 030001 Shanxi China; 5 Departments of Stomatology, The Sixed Hospital of Wuhan, Wuhan, Hubei China; 6 Departments of Stomatology, The First Hospital of Yulin, Yulin, Shaanxi China; 7 Departments of Stomatology, The First Affiliated Hospital of Sun Yat-Sen University, Guangzhou, Guangdong China

**Keywords:** Dentistry, Medical imaging, Materials science

## Abstract

This study aimed to compare MRI quality between conventional fast spin echo T_2_ weighted imaging (FSE T_2_WI) with periodically rotated overlapping parallel lines with enhanced reconstruction (PROPELLER) FSE T_2_WI for patients with various porcelain fused to metal (PFM) crown and analyze the value of PROPELLER technique in reducing metal artifacts. Conventional FSE T_2_WI and PROPELLER FSE T_2_WI sequences for axial imaging of head were applied in participants with different PFM crowns: cobalt-chromium (Co–Cr) alloy, pure titanium (Ti), gold–palladium (Au–Pd) alloy. Two radiologists evaluated overall image quality of section in PFM using a 5-point scale qualitatively and measured the maximum artifact area and artifact signal-to-noise ratio (SNR) quantitatively. Fifty-nine participants were evaluated. The metal crown with the least artifacts and the optimum image quality shown in conventional FSE T_2_WI and PROPELLER FSE T_2_WI were in Au–Pd alloy, Ti, and Co–Cr alloy order. PROPELLER FSE T_2_WI was superior to conventional FSE T_2_WI in improving image quality and reducing artifact area for Co-Cr alloy (17.0 ± 0.2% smaller artifact area, *p* < 0.001) and Ti (11.6 ± 0.7% smaller artifact area, *p* = 0.005), but had similar performance compared to FSE T_2_WI for Au–Pd alloy. The SNRs of the tongue and masseter muscle were significantly higher on PROPELLER FSE T_2_WI compared with conventional FSE T_2_WI (tongue: 29.76 ± 8.45 vs. 21.54 ± 9.31, *p* = 0.007; masseter muscle: 19.11 ± 8.24 vs. 15.26 ± 6.08, *p* = 0.016). Therefore, the different PFM crown generate varying degrees of metal artifacts in MRI, and the PROPELLER can effectively reduce metal artifacts especially in the PFM crown of Co-Cr alloy.

## Introduction

The porcelain fused to metal (PFM) is the conventional traditional method for fixed dentures in patients with dental defects or dentition defects and is especially used for single tooth restoration^1^. The PFM are valued for their visual appeal (they can match the color of the surrounding teeth and have similar visual properties to natural teeth), are extremely durable, and affordable. Also, these materials are not expensive and offer superior mechanical properties, and inertness compared with all other ceramic crowns^2,3^. At present, cobalt-chromium (Co-Cr) alloy, pure titanium (Ti) and gold–palladium (Au–Pd) alloy are the most conventional materials used for PFM crowns^2^.

Magnetic resonance imaging (MRI) is the most conventional head-neck clinical imaging technique used in clinical work mainly due to its non-ionizing radiation nature and superior soft-tissue image contrast^4,5^. However, MR image quality of the oral cavity and maxillofacial is often impaired by metallic dental restorations and implant-supported prostheses^6^. For example, the metal crown of PFM causes artifacts including signal-loss, signal-pileup, geometric distortion^6,7^, which can affect the visibilities of the anatomic structures near the PFM such as a tooth, periodontal space, tongue. The artifacts of metal implants depend on many factors, including the MRI hardware and room shielding, MRI software, sequence parameters, amount, shape, and material characteristics of used abutments and metal crowns. Though several studies have described the effect of material characteristics on MRI interpretation as the most significant among these factors^8,9^, few have addressed these problems in clinical situations. Therefore, identifying preferable material compositions of PFM crown and investigating methods to reduce or avoid metal artifacts in patients with PFM may improve individualized treatment and MRI scanning regimens^8^.

To address the decreased image quality due to metal implants, some optimized MRI scanning protocols were proposed to minimize the metal artifacts, such as using spin-echo or fast spin-echo sequences with long echo train lengths, a high bandwidth, thin section selection, and an increased matrix^10^. Recently, several MRI sequences were developed to reduce susceptibility artifacts, including view angle tilting (VAT), slice-encoding for metal artifact correction (SEMAC), multi-acquisition with variable resonance image combination (MAVRIC), ultrashort time-to-echo (UTE) and combinations of these techniques^11–13^. Furthermore, various deep learning-based approaches were developed to reduce metal artifacts, improve image quality, and even predict the missing information/regions in MR images affected by metal artifacts^14–16^. However, the clinical uses of these methods were restricted due to safety and quality control issues, as well as complex principles and higher demand for MRI equipment in hardware and software^17^.

Periodically rotated overlapping parallel lines with enhanced reconstruction (PROPELLER) combines a fast-spin echo (FSE) has been shown to be effective in decreasing motion artifact and suppressing flow artifacts after applying of contrast agent in the whole body^19–21^. The recent study confirmed that the PROPELLER sequence could decreases metallic artifacts apart from motion artifacts. The sequence was used to minimize the metallic artifacts and distortion near a metallic prosthesis in patients with hip metal work^22^. So, we hypothesize that the PROPELLER sequences would probably reduce metal artifacts of PFM crowns.

Therefore, we aimed to compare MR imaging quality in conventional FSE T_2_WI with PROPELLER FSE T_2_WI for patients with PFM of different metal crowns and investigate the value of the PROPELLER technique in reducing metal artifacts.

## Materials and methods

### Study population

This prospective study was approved by the local ethics committee (Second Hospital of Shanxi Medical University, Taiyuan, China). Written informed consent was obtained from all participants. All methods were performed in accordance with the relevant guidelines and regulations. Between July 2020 and March 2021, participants with single unit PFM crowns of three different metal materials scheduled to undergo a clinically indicated 1.5 T head MRI for known or suspected head pathology (cerebrovascular disease, tumor, infectious lesion) were enrolled in this study from the Department of Stomatology (Second Hospital of Shanxi Medical University) consecutively. Inclusion criteria included participants who had no metal fillings, implants, titanium plates or other metal materials except for PFM, had good compliance and were eligible for MRI. Exclusion criteria included space-occupying lesion in the oral cavity and maxillofacial regions or motion artifacts of MRI images. The participant characteristics (e g. age, sex, and material of metal crown) were collected. The material composition and properties of PFM crowns used in the study were: Co-Cr (material composition, Co 62%, Cr 28%, W 8.5%, Si 1.65%, Fe 0.5%, Mn 0.25%, C 0.1%; Coefficient of thermal expansion, 14.5 ± 0.5 × 10^-6^ K^-1^; Tensile strength, 525Mpa; Modulus of elasticity, 190,000), Ti (material composition, Ti 96%; Coefficient of thermal expansion, 14.3 ± 0.5 × 10^-6^ K^-1^; Tensile strength, 552Mpa; Modulus of elasticity, 105,000), and Au–Pd (material composition, Au 74, Pt 8.5, Pd 5.4, Ag 8.98, In 1.9; Coefficient of thermal expansion, 9.6 ± 0.5 × 10^-6^ K^−1^; Tensile strength, 441Mpa; Modulus of elasticity, 82,000).

### Image acquisition

All MRI data were acquired on a 1.5 T MR scanner (Signa; GE Healthcare, Waukesha, Wis) with an 8-channel head and neck coil. Each participant was placed in the supine position, and underwent head MRI scan. The MRI sequences consisted of axial FSE T_2_WI, axial PROPELLER FSE T_2_WI, axial FSE T1WI, axial diffusion weighted imaging (DWI) and sagittal FSE T1WI. Parameters of axial FSE T_2_WI were as follows: repetition time (TR)/echo time (TE), 3000/113 ms; Field of View (FOV), 240 × 240mm^2^; slice thickness/gap, 6/1 mm; the number of excitations (NEX), 2; Echo-train length (ETL), 19; matrix size, 352 × 352; Axial PROPELLER was optimized by selecting a bandwidth to minimize TE with the following parameters: TR/TE, 3000/112 ms; NEX, 2; ETL, 32; FOV, matrix size, slice thickness and orientation were matched to T_2_WI. All sequences used 2D acquisition.

### Image analysis

The image quality of conventional axial FSE T_2_WI and axial PROPELLER FSE T_2_WI were evaluated quantitatively and qualitatively using our institution´s picture archiving and communication system (PACS) workstation (Advantage Windows Workstation 4.6; GE Healthcare, Madison, WI, USA). The images were independently reviewed and scored by two radiologists (X.X. and X. X.), with 5 and 10 years of experience in head and neck MRI, respectively. All images were deidentified and evaluated in a blinded and randomized fashion with respect to the method of image acquisition.

### Qualitative image analysis

Visualizations of the anatomic structures around PFM were evaluated by two radiologists using a 5-point scale in all image sets as described before^13,23^. The visibilities of four anatomic structures near the PFM including visualization of the periodontal space, the tooth, the tongue, and bone (maxilla or mandible) in the MR images were graded as follows: grade 1 indicated the worst quality for interpretation where the anatomic structures around PFM were barely delineated; grade 2 indicated that 25% of the above structures were visible; grade 3, visualization of 50% of the above structures; grade 4, visualization of 75% of the above structures; and grade 5, none of the four anatomical structures around the PFM were affected by artifacts.

### Quantitative image analysis

The artifact was defined as areas of signal void pileup or geometric distortion^24^. First, the plane with the maximal artifact were determined, then the maximum areas of metal artifacts were outlined and measured by two radiologists jointly using the picture archiving and communications system workstation (Advantage Windows Workstation 4.6; GE Healthcare, Madison, WI, USA). All images analyzed by the radiologists were set on the same window widths/levels which generated by the workstation automatically. The artifact area reduction rate of PROPELLER FSE T_2_WI image were calculated as the difference between PROPELLER FSE T_2_WI image artifact area and conventional FSE T_2_WI image artifact area divided by conventional FSE T_2_WI image artifact area. Next, in the same plane and display conditions, SNRs were calculated through manual signal intensity measurements of tongue, fat, muscle on conventional FSE T_2_WI and PROPELLER FSE T_2_WI data sets^25,26^. Signal intensity values and the standard deviation of room air were obtained by using the Advantage Workstation. Tongue was measured in the center regions of tongue tissue, fat was measured in the lateral subcutaneous adipose tissue of artifacts, while Muscle was measured in the masseter muscle ipsilateral to the artifacts. SNRs were defined as SI_ROI_/SD_background_^27^, where SI_ROI_ is the mean signal intensity of the ROI of the respective tissue and SD_background_ is the standard deviation of the background signal. The ROIs were 0.5cm^2^ for tongue, fat, muscle and background. The background noise was measured with 4 ROIs of 0.5cm^2^ that were positioned in room air at the level of the anterior, posterior, left and right close to the skin surface of the head. Positioning of these ROIs were identical in conventional FSE T_2_WI and PROPELLER FSE T_2_WI images to minimize individual variations for sequence comparison.

### Statistical analysis

Statistical analysis was performed using SPSS 25.0 software (SPSS, Chicago, IL, USA). Interrater agreement for qualitative scores was assessed by Cohen’s weighted kappa (*κ*) and was interpreted as follows: Poor correlation (*κ* < 0.20); Fair correlation (*κ* = 0.21–0.40); Moderate correlation (*κ* = 0.41–0.60); Good correlation (*κ* = 0.61–0.80); and excellent correlation (*κ* = 0.81–1.00). The image quality scores and artifact areas of different metal crowns were compared using post-hoc analysis's Friedman test. The image quality scores, artifact areas and SNR of all conventional FSE T_2_WI and PROPELLER FSE T_2_WI were compared using a two-sample Wilcoxon test. Results were provided as mean ± standard deviation (SD). A P value < 0.05 was considered to be statistically significant.

## Results

### Patient data

A total of 64 participants with single unit PFM crowns underwent MRI in the head. One participant with maxillofacial space-occupying lesion, two participants with tumor of tongue and two participants with motion artifacts were excluded. The final study sample consisted of 59 participants (24 males and 35 females; mean age ± SD, 63 ± 5.2 years; age range,35–70 years old). There were 21 participants with the metal crown of Co-Cr alloy in PFM, 20 with the metal crown of Au–Pd alloy in PFM, and 18 participants with the metal crown of Ti in PFM.

### Inter-reader variability

There were good agreements between readers for scoring image quality of Co–Cr (*κ* = 0.80) and Au–Pd (*κ* = 0.73) in conventional FSE T_2_WI, with the excellent agreement (κ = 0.84) for the image quality of Ti. PROPELLER FSE T_2_WI demonstrated good agreements between readers for the image quality of Co–Cr (*κ* = 0.77), Au–Pd (*κ* = 0.78), and Ti (*κ* = 0.67). The interreader variability parameters are summarized in Table 1.

**Table 1:** Interrater agreement for image qualitative scores in conventional FSE T_2_WI and PROPELLER FSE T_2_WI sequences.

Material composition	Conventional FSE T_2_WI	PROPELLER FSE T_2_WI
Co–Cr (n = 21)	0.80 (0.63–0.90)	0.77 (0.57–0.89)
Ti (n = 18)	0.84 (0.69–0.92)	0.67 (0.41–0.82)
Au–Pd (n = 20)	0.73 (0.44–0.87)	0.78 (0.62–0.94)

Interreader variability is statistically significant (*P* < 0.001); FSE T_2_WI, fast spin echo T_2_ weighted imaging; PROPELLER FSE T2WI, periodically rotated overlapping parallel lines with enhanced reconstruction fast spin echo T2 weighted imaging; Co–Cr, cobalt-chromium; Ti, titanium; Au–Pd, gold–palladium.

### Comparison of artifacts between conventional FSE T_2_WI and PROPELLER FSE T_2_WI for three different material compositions

**Figure 1:**
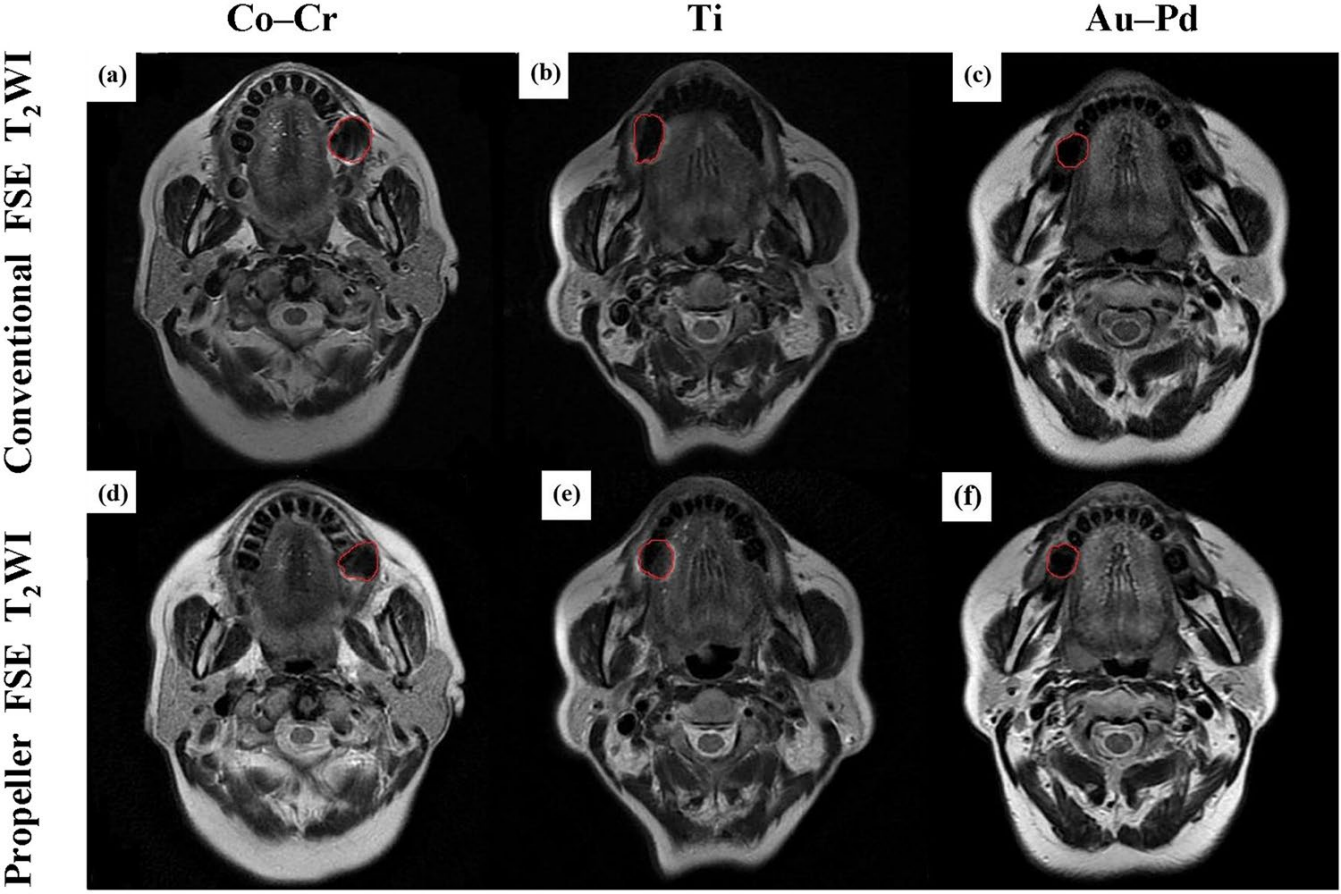
Images of conventional FSE T_2_WI (**a**–**c**) and PROPELLER FSE T_2_WI (**d**–**f**) in different participants. Images of the metal crown of Co-Cr alloy in participant 1 (**a**,**d**); Images of the metal crown of pure Ti in participant 2 (**b**,**e**); Images of the metal crown of Au–Pd alloy in participant 3 (**c**,**f**).

**Figure 2:**
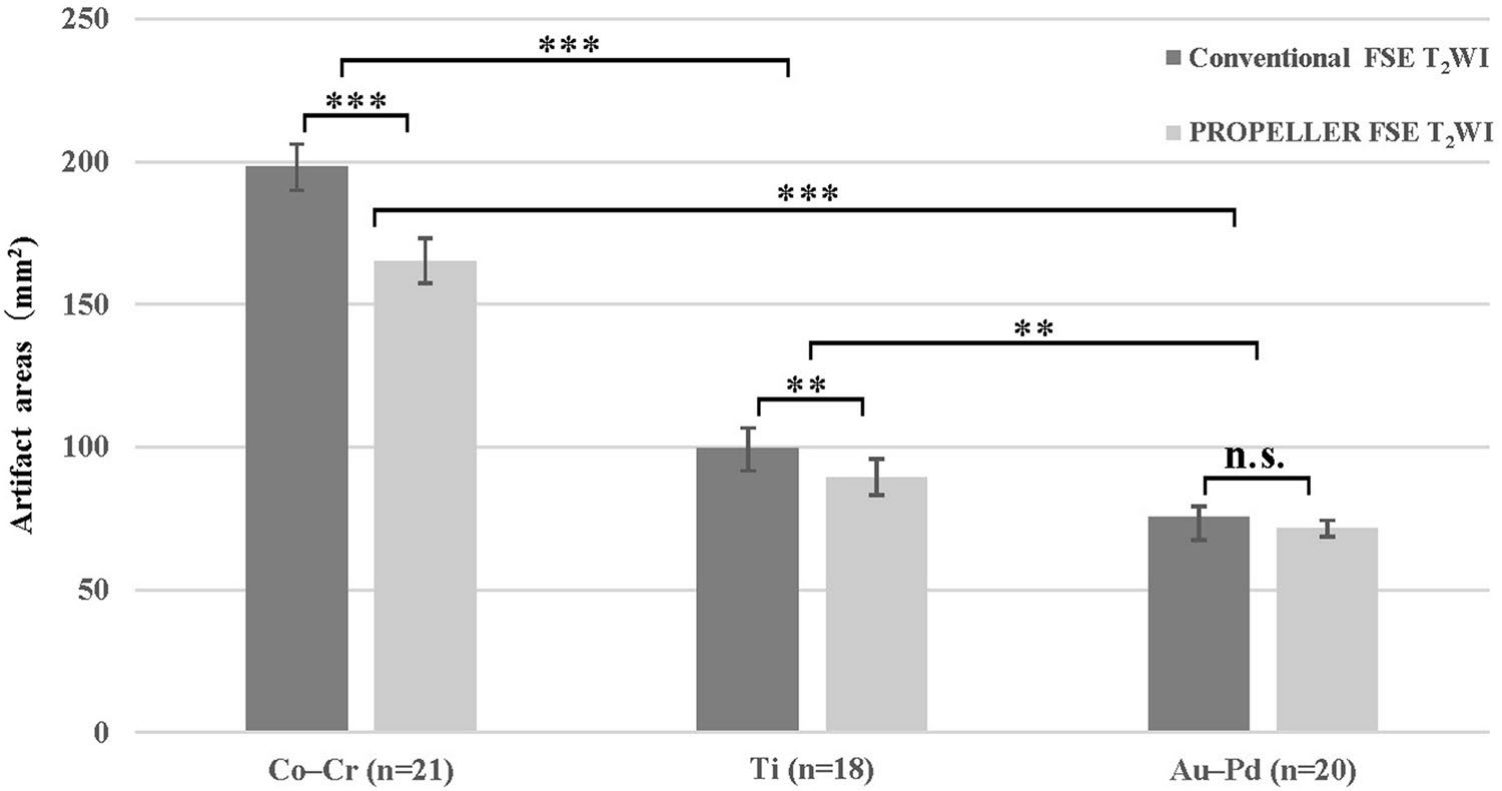
Comparison of artifact areas of conventional FSE T_2_WI and PROPELLER FSE T_2_WI sequences caused by the different PFM crowns. n.s, not significant; **, *P* < 0.01; ***, *P* < 0.001.

Artifacts caused by three different PFM crowns can be clearly identified on both conventional FSE T_2_WI and PROPELLER FSE T_2_WI (Fig. 1). The image quality of Au–Pd was significantly better than those of Ti and Co-Cr in conventional FSE T_2_WI (reader 1 (score ± SD): Au–Pd 3.5 ± 0.1 vs. Ti 1.6 ± 0.2 vs. Co-Cr 1.4 ± 0.1, *p* = 0.028; reader 2: Au–Pd 3.6 ± 0.1 vs. Ti 1.6 ± 0.2 vs. Co-Cr 1.4 ± 0.1, *p* = 0.006) and PROPELLER FSE T_2_WI (reader 1 (score ± SD): Au–Pd 3.6 ± 0.2 vs. Ti 1.8 ± 0.2 vs. Co-Cr 1.7 ± 0.1, *p* = 0.021; reader 2: Au–Pd 3.6 ± 0.2 vs. Ti 1.9 ± 0.2 vs. Co-Cr 1.7 ± 0.2, *p* = 0.002). Moreover, Ti had a slightly higher image quality score than Co-Cr in both sequences, however these differences were not statistically significant (reader 1: *p* = 0.36, *p* = 1.0; reader 2: *p* = 0.42, *p* = 0.84, respectively). In addition, the artifact areas caused by the metal crown of Co-Cr, Ti and Au–Pd were significantly different from each other in conventional FSE T_2_WI (Au–Pd 75.4 ± 3.8mm^2^ vs. Ti 99.7 ± 7.0mm^2^ vs. Co-Cr 198.1 ± 8.1mm^2^, *p* < 0.001) and PROPELLER FSE T_2_WI (Au–Pd 71.4 ± 2.9mm^2^ vs. Ti 89.4 ± 6.3mm^2^ vs. Co-Cr 165.3 ± 7.9mm^2^, *p* < 0.001, Fig. 2). The smallest artifact area was observed for Au–Pd in both sequences.

The conventional FSE T_2_WI sequence and PROPELLER FSE T_2_WI sequence differed significantly in image quality score and artifact area for Co–Cr and Ti, but not for the Au–Pd. PROPELLER FSE T_2_WI exhibited significantly better image quality than conventional FSE T_2_WI for Co–Cr (reader 1 (score ± SD): 1.4 ± 0.1 vs. 1.8 ± 0.1, *p* < 0.001; reader 2: 1.4 ± 0.1 vs. 1.8 ± 0.2, *p* < 0.001) and Ti (reader 1 (score ± SD): 1.6 ± 0.2 vs. 1.8 ± 0.2, p < 0.001; reader 2: 1.6 ± 0.2 vs. 1.9 ± 0.2, *p* < 0.001). There was a significant decrease of 17.0 ± 0.2% in artifact area in PROPELLER FSE T_2_WI compared with conventional FSE T_2_WI for the Co–Cr (198.1 ± 8.1mm^2^ vs. 165.3 ± 7.9mm^2^, *p* < 0.001), and 11.6 ± 0.7% for the Ti (99.7 ± 7.0mm^2^ vs. 89.4 ± 6.3 mm^2^, *p* = 0.005, Fig. 2). The SNRs of the tongue and masseter muscle were significantly higher on PROPELLER FSE T_2_WI compared with conventional FSE T_2_WI (tongue: 29.76 ± 8.45 vs. 21.54 ± 9.31, *p* = 0.007; masseter muscle: 19.11 ± 8.24 vs. 15.26 ± 6.08, *p* = 0.016), whereas there was no significant difference of SNR for fat (113.39 ± 30.42 vs. 104.72 ± 26.45, *p* = 0.315;). The results are summarized in Tables 2 and 3.

**Table 2:** Comparison of image quality scores of different metal crowns in conventional FSE T_2_WI and PROPELLER FSE T_2_WI sequences.

Material composition	Conventional FSE T_2_WI	PROPELLER FSE T_2_WI	*P* value^a^
**Reader 1 (mean ± SD)**			
Co–Cr (n = 21)	1.4 ± 0.1	1.7 ± 0.1	< 0.001
Ti (n = 18)	1.6 ± 0.2	1.8 ± 0.2	< 0.001
Au–Pd (n = 20)	3.5 ± 0.1	3.6 ± 0.2	0.350
*P* value^b^	0.028	0.021	–
**Reader 2 (mean ± SD)**			
Co–Cr (n = 21)	1.4 ± 0.1	1.7 ± 0.2	< 0.001
Ti (n = 18)	1.6 ± 0.2	1.9 ± 0.2	< 0.001
Au–Pd (n = 20)	3.6 ± 0.1	3.6 ± 0.2	0.416
*P* value^b^	0.006	0.002	–

FSE T_2_WI, fast spin echo T_2_ weighted imaging; PROPELLER FSE T_2_WI, periodically rotated overlapping parallel lines with enhanced reconstruction fast spin echo T_2_ weighted imaging; Co–Cr, cobalt-chromium; Ti, titanium; Au–Pd, gold–palladium. ^a^Two-sample Wilcoxon test. Significant *P*-values were < 0.05. ^b^Friedman test. Significant *P*-values were < 0.05.

**Table 3:** Comparison of artifact areas of different metal crowns in conventional FSE T_2_WI and PROPELLER FSE T_2_WI sequences(mm^2^).

Material composition	Conventional FSE T_2_WI	PROPELLER FSE T_2_WI	*P* value^a^	Artifact reduction
Co–Cr (n = 21)	198.1 ± 8.1	165.3 ± 7.9	< 0.001	17.0 ± 0.2%
Ti (n = 18)	99.7 ± 7.0	89.4 ± 6.3	0.005	11.6 ± 0.7%
Au–Pd (n = 20)	75.4 ± 3.8	71.4 ± 2.9	0.057	5.1 ± 0.5%
*P* value^b^	< 0.001	< 0.001	–	< 0.001

FSE T_2_WI, fast spin echo T_2_ weighted imaging; PROPELLER FSE T_2_WI, periodically rotated overlapping parallel lines with enhanced reconstruction fast spin echo T_2_ weighted imaging; Co–Cr, cobalt-chromium; Ti, titanium; Au–Pd, gold–palladium. ^a^Two-sample Wilcoxon test. Significant *P*-values were < 0.05.^b^Friedman test.

## Discussion

PFM restorations are increasingly used in prosthetic dentistry^28^. Dental MRI offers radiation-free and high-resolution in vivo imaging of the teeth, jaw and adjacent soft tissue. However, image assessment may be distorted by artifacts due to metallic dental restorations. Prior studies have demonstrated that the PROPELLER sequence could reduce metal artifacts in patients with a metal implant for orthopedic and neurosurgical applications^19,23^. Our study has further shown that PROPELLER FSE T_2_WI may significantly improve imaging quality and reduce artifact areas compared to the conventional FSE T_2_WI sequence in dental MR imaging especially in the PFM crown of Co-Cr alloy.

There is an increasing demand for PFM in patients with dental defects or dentition defects^29^. In the future, radiologists will be required to select appropriate MRI sequences and parameters which could reduce metal artifacts caused by metal crowns in PFM, because high quality MR images was benefit of clinicians for diagnosis of diseases in head and oromaxillo-facial region. Therefore, the understanding about causes of artifacts related to metal implants on MR images would be beneficial to dentists making individualized regimens^6^. Previous studies on the effect of different dental metal materials on MRI artifacts were mostly based on in vitro phantoms. Our study investigated the effects of artifacts caused by various porcelain fused to metal (PFM) crowns on patients' head MRI, which has more clinical applicability. Our data suggested that the PFM crown of Co-Cr alloy produces the more metal artifacts compared to the pure Ti and the gold–palladium alloy in the conventional FSE T_2_WI and PROPELLER FSE T_2_WI sequence. The reasons are most likely due to the specific ferromagnetic compositions of these alloys. Cobalt and chromium are ferromagnetic metals, they distort local magnetic fields, causing large artifacts that make image interpretation impossible. Titanium itself has ferromagnetic properties but has a lower magnetic susceptibility. Although gold is a diamagnetic substance, gold alloys contain traces of other ferromagnetic metals could also explain the ability to degrade MRI images^8,30^. Nevertheless, some studies showed that high gold-content alloys and pure Ti materials produce more artifacts^31,32^. The probable reasons were (1) The materials used probably come from different manufacturers with varied standards for material processing^33^. (2) MRI scanners, imaging parameters and experimental methods used in different studies are different. (3) The research objects were not unified, including dental implants, orthodontic devices, embedded phantoms etc^34^. Therefore, it is necessary to formulate unified experimental criteria for accurately evaluating the effects of different dental materials on MRI metallic artifacts.

The previous studies showed that the spin-echo (SE) sequence significantly reduces the susceptibility artifact compared with the gradient-echo (GRE) sequence, yet, this still did not meet the expected standards. Furthermore, advances in MR sequence (e.g., VAT, SEMAC, MAVRIC, UTE) and serious deep learn-based methods now allow significantly improved image quality in the presence of ferromagnetic materials^11–16^. The PROPELLER sequence has the advantages of mature technology, imaging easily, high SNR, and can be combined with various sequences such as FLAIR, DWI. It can be widely used in clinical practice compared with other complex techniques to reduce artifacts. Most previous studies applied it to reduce motion artifacts^19–21^. The recent study confirms that the PROPELLER could decreases both artifact and distortion in patients with hip metalwork^22^. Our qualitative and quantitative studies confirmed that PROPELLER FSE T_2_WI significantly improves imaging quality and reduces artifact areas compared to the conventional FSE T_2_WI sequence in patients with PFM. Meanwhile PROPELLER FSE T_2_WI had the best efficiency on reducing metal artifacts of cobalt-chromium alloy compared with the other two kinds of materials in our study. These findings can be attributed to (1) PROPELLER’s unique radial k-space acquisition sequencing: PROPELLER fills the k-space with a rotating stripe, so that all the stripe have overlapping k-space center. The overlapped k-space center region may be used to estimate the k-space phase differences caused by metal when different stripes are acquired, which diminishes artifact in the phase-encoding direction^32^. (2) Susceptibility effects primarily affect T_2_*signal decay, by inducing local distortions in the static magnetic field. Therefore, the PROPELLER sequence refocuses T_2_ using a spin-echo pulse prior to each readout and removes the distorted T_2_ * signal component from the NMR signal to reduce the subsequent image distortion caused by magnetic susceptibility^22^. Similar to the previous results^19,35^, our study showed the SNRs of the tongue and masseter muscle were significantly higher on PROPELLER FSE T_2_WI compared with conventional FSE T_2_WI. This was mainly because PROPELLER oversampled data at the center of the k-space which determined the SNR and contrast of images^35^.

Our study has several limitations. First, all MRI examinations were performed at 1.5 T, and we did not obtain sagittal and coronal images. Because the level of metallic susceptibility artifact in output images is directly related to field strength, we anticipate that there might be larger artifact when scanning at 3 T. For the sake of clinical imaging diagnosis, further research is necessary to combine axial, sagittal and coronal images to study the ability of PROPELLER to reduce metal artifacts in patients with PFM. Second, although our study selected adult participants whose PFM was closed to same size, the shape of PFM and the porcelain in PFM may be varied, which may lead to bias. Third, in the qualitative evaluation, we only evaluate the effect of artifacts on the overall image of conventional head MRI, and a separate score for each anatomical structure has not been performed. Fourth, we only studied the effect of PROPELLER technology in reducing artifacts of metal copying in the single unit PFM crowns, the applications of PROPELLER technology to metal artifact reduction in multiple unit PFM crowns and other oral fields such as implant prosthesis and orthodontics will be performed in the next study. Finally, the sample size was small. Increasing the sample size would have increased the statistical power of the study.

In conclusion, the different PFM crown generates varying degrees of metal artifact areas in MR imaging. The PROPELLER sequence can effectively reduce metal artifacts in dental MR imaging especially in the PFM crown of Co-Cr alloy. These findings could add value to the clinical management and MRI examination planning in patients with PFM. 

## Data Availability

Te datasets generated during and/or analysed during the current study are available from the corresponding author on reasonable request.
